# Fragmentation Study, Dual Anti-Bactericidal and Anti-Viral Effects and Molecular Docking of Cobalt(III) Complexes

**DOI:** 10.3390/ijms21218355

**Published:** 2020-11-07

**Authors:** Laísa de P. Fernandes, Júlia M. B. Silva, Daniel O. S. Martins, Mariana B. Santiago, Carlos H. G. Martins, Ana C. G. Jardim, Guedmiller S. Oliveira, Marcos Pivatto, Rafael A. C. Souza, Eduardo de F. Franca, Victor M. Deflon, Antonio E. H. Machado, Carolina G. Oliveira

**Affiliations:** 1 Instituto de Química, Universidade Federal de Uberlândia, Uberlândia 38400-902, MG, Brazil; laisadepaulla@hotmail.com (L.d.P.F.); guedmiller@ufu.br (G.S.O.); mpivatto@gmail.com (M.P.); rafasouza27@hotmail.com (R.A.C.S.); 2 Instituto de Ciências Biomédicas, Universidade Federal de Uberlândia, Uberlândia 38408-100, MG, Brazil; juliabraz.contato@outlook.com (J.M.B.S.); danielosmartins@gmail.com (D.O.S.M.); mari.brentini@hotmail.com (M.B.S.); carlos.martins2@ufu.br (C.H.G.M.); jardim@ufu.br (A.C.G.J.); 3 Laboratório de Cristalografia e Química Computacional, Instituto de Química, Universidade Federal de Uberlândia, UFU, Uberlândia 38408-100, MG, Brazil; eduardofranca@ufu.br; 4 Instituto de Química de São Carlos, Universidade de São Paulo, São Carlos 13566-590, SP, Brazil; deflon@usp.br; 5 Laboratório de Fotoquímica e Ciências dos Materiais, Instituto de Química, Universidade Federal de Uberlândia, Uberlândia 38400-902, MG, Brazil; aehmachado@gmail.com; 6 Unidade Acadêmica Especial de Física, Programa de Pós-Graduação em Ciências Exatas e Tecnol., Universidade Federal de Catalão, Catalão 75705-220, GO, Brasil

**Keywords:** cobalt(III) complexes, biological application, mass spectrometry, molecular docking

## Abstract

Considering our previous findings on the remarkable activity exhibited by cobalt(III) with 2-acetylpyridine-*N(4)*-R-thiosemicarbazone (Hatc-R) compounds against *Mycobacterium tuberculosis*, the present study aimed to explored new structure features of the complexes of the type [Co(atc--R)_2_]Cl, where R = methyl (Me, **1**) or phenyl (Ph, **2**) (^13^C NMR, high-resolution mass spectrometry, LC–MS/MS, fragmentation study) together with its antibacterial and antiviral biological activities. The minimal inhibitory and minimal bactericidal concentrations (MIC and MBC) were determined, as well as the antiviral potential of the complexes on chikungunya virus (CHIKV) infection in vitro and cell viability. [Co(atc-Ph)_2_]Cl revealed promising MIC and MBC values which ranged from 0.39 to 0.78 µg/mL in two strains tested and presented high potential against CHIKV by reducing viral replication by up to 80%. The results showed that the biological activity is strongly influenced by the peripheral substituent groups at the *N*(4) position of the atc-R^1−^ ligands. In addition, molecular docking analysis was performed. The relative binding energy of the docked compound with five bacteria strains was found in the range of −3.45 and −9.55 kcal/mol. Thus, this work highlights the good potential of cobalt(III) complexes and provide support for future studies on this molecule aiming at its antibacterial and antiviral therapeutic application.

## 1. Introduction

The clinical achievement of inorganic drugs, such as cisplatin and its derivates chemotherapeutics agents, has significantly improved the study of metal ions on the biological system for the treatment of several diseases [1,2,3]. The ability to form complexes with different coordination numbers, the geometries, the varied oxidation number and the possibility to bind to biomolecules are some features that prove the potential of metal ions as interesting means to explore innovation in drug discovery. Over the years, many transition metal complexes of Cu, Ni, Zn, Co, and Pd have been reported to present remarkable biological activity in a considerable number of illnesses [4,5,6,7,8].

In the bioinorganic chemistry field, besides the strong research on active drugs containing essential metals including Fe [9], Zn [8,10], Cu [11], Co [12], and Mg [13], among others, metallodrugs based on those metal ions have not successfully advanced in clinical trials so far. Moreover, a great advantage of potential drugs containing an essential metal would be the lower levels of toxicity on healthy cells. As vital metal ions, they play essential roles, being responsible for transporters, carriers, binding proteins and cofactors of a wide range of metalloproteins [14]. Cobalt, an essential trace element present in human body, is the main component of cobalamin, vitamin B12 [15]. The Co(III) ion in cobalamin is stabilized by a chelating tetradentate macrocycle in which four nitrogen donor atoms are located in the equatorial plane, and the axial positions are filled by adenosyl residue in an octahedral geometry [16]. This important metal ion is even involved in essential biological roles such as amino acid and fatty acid metabolism and hematopoiesis [17].

Interestingly, to the best of our knowledge, only one cobalt complex containing Schiff base as a ligand, named bis(2-methylimidazole)[(bis(acetylacetone)(ethylenediimine)]-cobalt(III), known as CTC-96 (Doxovir) [18], has recently been tested in a phase II clinical trial for anti-viral treatment [6]. Doxovir presents antiviral biological proprieties for herpes simplex virus (HSV) and for the treatment of two viral eye infections (adenoviral conjunctivitis and ophthalmic herpetic keratitis) [19]. In vitro and in vivo studies have explained the activity of CTC-96 against HSV by interactions of the complex with the viral envelope membrane, which consequently prevent the entry of the virus into the cells, but the exact mechanism is not fully understood [20].

Despite the extensive investigation on metal complexes as chemotherapeutic candidates, only a small number of cobalt(III) complexes are known as inorganic pharmaceuticals when compared to other metals (platinum and gold, for example). In view of the constant evolution of microorganism resistance, virus mutations and the limited existence of potential drugs with antibacterial and antiviral activity, it is necessary to develop new therapeutic alternatives. In this context, thiosemicarbazones Schiff bases are a recognized class of bioactive molecules with antimicrobial and antiviral purposes [21,22]. It is well established that the complexation of thiosemicarbazone ligands to metal ions can show considerable efficacy to improve biological activity [3,7,23,24]. Furthermore, this class of ligands has shown reduced toxic effects when chelated to metal ions [23]. In addition, the number of reports on the coordination chemistry modifications of thiosemicarbazone ligands has appeared to be associated with a rich variety, which is directly correlated to their biological activity [25].

Researchers have described cobalt complexes as promising anti-bacterial agents [26], and few studies show its antiviral potential [21]. Additionally, the activity of Co(III) with Schiff base 2-acetyl-piridine-*N*(4)-R-thiosemicarbazone (R = hydrogen, methyl, ethyl, cyclohexyl and phenyl) has been described in the literature. In addition to the satisfactory antibacterial activity of this type of complex on *Mycobacterium tuberculosis* H37Rv (ATCC 27294) bacteria with a minimal inhibitory concentration (MIC) of less than 10 µg/mL [27], the toxicity evaluated on normal cells (VERO and J774A.1) discovered the phenyl derivative complex as the highest selective agent (selective index of 17). The authors noted that the selectivity was highly dependent on the bulkiness of the R group, which probably increases the relative lipophilicity of the molecule. However, there are still few studies on the activity of Co(III) thiosemicarbazonate-containing compounds. Thus, the exploration of the potential of octahedral cationic cobalt(III) complexes with Schiff bases as inorganic pharmaceuticals seemed to be worthwhile.

Based on this, the aim of this study was to synthesize a known Schiff base Co(III) complex to design new structural experimental studies together with the antibacterial and antiviral activity, as well as further investigation of the interactions between the complex and bacteria through molecular docking analysis. Thus, this study is expected to provide new structural information and biological insights of cobalt(III) thiosemicarbazone-based compounds, encouraging the investigation of this substance as an alternative treatment for dental bacteria and chikungunya virus.

## 2. Results and Discussion

### 2.1. Structural Characterization

**Scheme 1:**
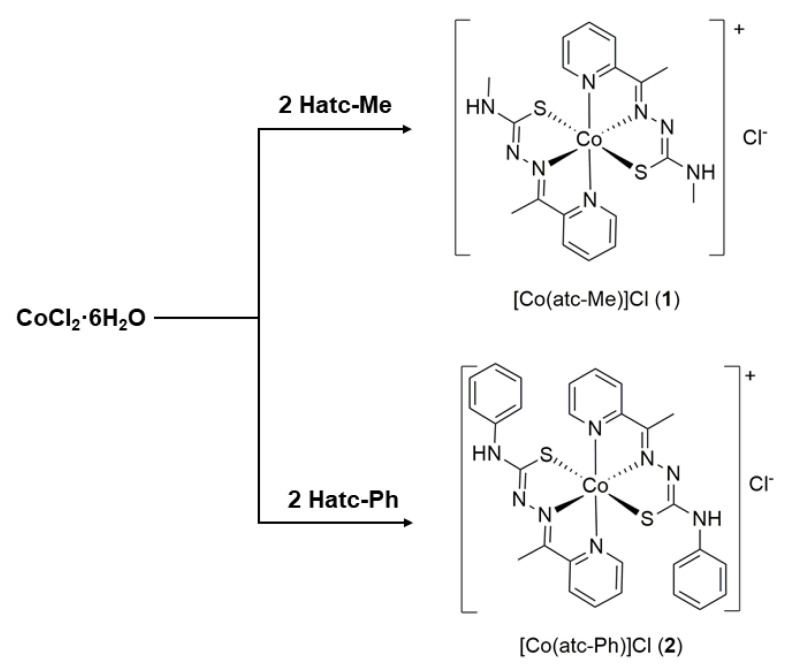
Synthesis of the cobalt(III) complexes.

Reaction of thiosemicarbazone ligands Hatc-R (R = methyl or phenyl) with cobalt(II) chloride hexahydrate precursor in ethanolic solution resulted in the products [Co(atc-Me)_2_]Cl (**1**) and [Co(atc-Ph)_2_]Cl (**2**) (Scheme 1). The product was isolated as a brown precipitate and separated by vacuum filtration after reduction the solution volume. The identity of the complexes was confirmed predominant by ^1^H, ^13^C NMR, LC–MS/MS and conductivity measurements, which confirmed the electrolytes 1:1. The results are in accordance with the oxidation of cobalt(II) center to cobalt(III) upon the coordination due to the greater stabilization of the oxidation state III caused by chelating binders [16]. Both complexes are very soluble in methanol and ethanol solutions, while complex **1** is water soluble. Additional and unprecedented characterization techniques, such as ^13^C NMR, LC–MS, and MS/MS, were employed for the Co(III) complexes prepared here to better understand the complexes’ behavior in solution.

The ^13^C NMR spectroscopic data for complexes **1** and **2** recorded in DMSO-*d*_6_ presented the expected signals, and the results are showed in Table S1. Upon coordination, a downfield shift around 4 ppm is observed for the C=N signals, while the signal for carbon in C=S fragment appears at 178.2 for complex **1** (Figure S1) and 175.5 ppm for **2** (Figure S2), which is in agreement with the *N,N,S* coordination and thioenolization of the C=S units of the thiourea and thiosemicarbazone moieties. The observed shifts are consistent with the *N,N,S* coordination of the (atc-R)^−^ ligands.

**Figure 1:**
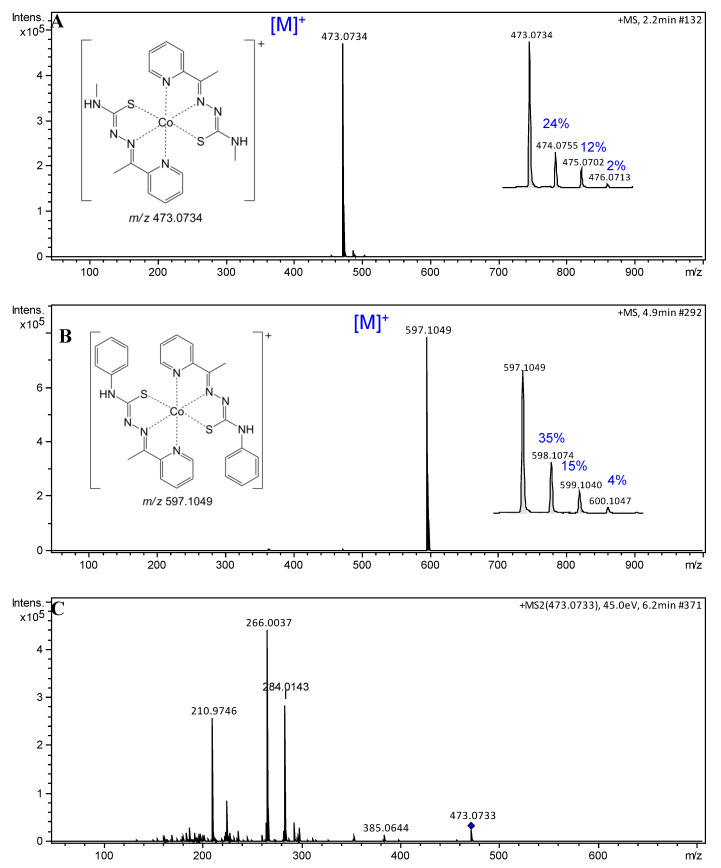

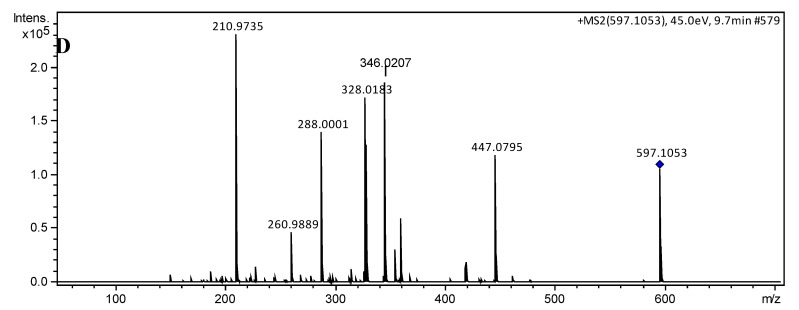
Electrospray ionization (ESI) mass spectra of cobalt(III) complexes **1** and **2**. (**A**) (+)-high-resolution electrospray ionization mass spectra (HRESIMS) spectrum of **1**, *m*/*z* 473.0734 [M]^+^ (calcd. for C_18_H_22_CoN_8_S_2_, 473.0735, Δ –0.21 ppm); (**B**) (+)-HRESIMS spectrum of **2**, *m*/*z* 597.1049 [M]^+^ (calcd. for C_28_H_26_CoN_8_S_2_, 597.1048, Δ 0.17 ppm); (**C**) ESI–MS/MS spectrum of the molecular ion *m*/*z* 473.0733; and (**D**) ESI–MS/MS spectrum of the molecular ion *m*/*z* 597.1053.

Information of interest is the structure–property relationship studies regarding the lipophilicity [28]. This factor represents the extent to which a drug and/or a substance prefers a hydrophilic or a lipophilic medium. A second important aspect is the different polarities that can suggest the behavior of a compound in biologic environments and even affect the cellular uptake [29,30]. A simple method of assessing the lipophilicity of two compound is to analyze their retention times (*t*_R_) in a chromatographic analysis [31]. Thus, compounds **1** and **2** were analyzed by LC–MS, and structural identifications were carried out through mass spectra analysis. Compound **1** (methyl derived) was identified as a majority signal at *t*_R_ 4.8 min and showed the charged complex ion peak [M]^+^ at *m*/*z* 473. Due to the presence of a phenyl group, complex **2** presented a higher retention time at *t*_R_ 7.7 min and showed a charged complex ion peak [M]^+^ at *m*/*z* 597. Thus, among them, complex **2** was more lipophilic. Both compounds showed high purity grade, determined to be 91 and 96% for **1** and **2**, respectively, according to LC–MS measurement (Figures S3 and S4, Supplementary Materials). Additionally, mass spectra of cobalt complexes are characterized by the presence of a clear isotopic pattern due to the fact that cobalt is a monoisotopic element ^59^Co (100%), with the nuclide abundance in parentheses [32]. High-resolution mass spectra of compounds **1** and **2** were recorded in the positive mode, and the obtained data confirm the established patterns (Figure 1A,B).

**Figure 2:**
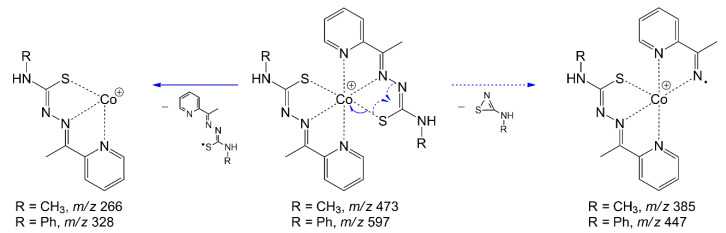
Fragmentation pathway proposed for **1** (*m*/*z* 473) and **2** (*m*/*z* 597) analyzed by (+)-ESI–MS/MS.

The charged complex ions were observed at *m*/*z* 473.0734 [M]^+^ (**1**) and 597.1049 [M]^+^ (**2**), in agreement with calculated values for C_18_H_22_CoN_8_S_2_ (473.0735; Δ −0.21 ppm) and C_28_H_26_CoN_8_S_2_ ( 597.1048; Δ 0.17 ppm), respectively. Collision-induced dissociation (CID) experiments (MS/MS) with an increasing collisional energy (5 to 50 eV) using N_2_ as collision gas under the selected ions at *m*/*z* 473 [M]^+^ (1) and 597 [M]^+^ (2) showed a similar fragmentation pathway for both complexes (Figure 2), which can be seen in the mass spectra with collisional energy of 45 eV (Figure 1C,D). When these signals were subjected to MS/MS analysis (Figure 1C,D), the fragment ions at *m*/*z* 266 and 328 arose, respectively, and were attributed to homolytic cleavage between the sulfur and the metal, reducing it to Co(II). Neutral losses of 88 or 150 amu produced fragment ions at *m*/*z* 385 and 447 for **1** and **2**, respectively, and were proposed for neutral elimination of a strained heterocyclic thiozone ring through homolytic cleavages between S–Co and N–N chemical bonds, according to the mechanism pathway proposed. Furthermore, MS/MS analysis suggested that the complexes have high stability after observing the fragments at high energies only.

### 2.2. Stability Studies

The stability of complexes **1** and **2** was monitored by ^1^H NMR in DMSO-*d*_6_ solution for a 24 h period. The spectra remained unchanged during the assay time, showing that the compound does not hydrolyze under such conditions (Figure S5).

### 2.3. Biological Activity Studies

Many efforts in bioinorganic chemistry field have been dedicated to the development of new agents based on a variety of metal ions compounds [33]. These metal-based compounds normally show promising activity against viruses [34], bacteria [35] and on cancer cell lines [36]. Due to the remarkable biological proprieties of thiosemicarbazone and the lower number of inorganic drugs on clinical trials, we believed that it was worth evaluating the antibacterial and antiviral activity of the cobalt(III) compounds developed in this work. As the adequate purity and stability in solution were certified, the compounds were submitted to the biological tests. Thus, the activity of the free ligands HL^R^ and their cobalt complexes was evaluated against the dental caries pathogens and on chikungunya virus (CHIKV).

#### 2.3.1. Antibacterial Activity

The compounds were evaluated in relation to their antibacterial activity. Table 1 shows the MIC and minimal bactericidal concentration (MBC) values determined for the free ligands Hatc-Me and Hatc-Ph, the complexes [Co(atc-Me)_2_]Cl (**1**) and [Co(atc-Ph)_2_]Cl (**2**), and the starting material for the synthesis CoCl_2_ꞏ6H_2_O (**3**) against seven cariogenic bacteria. The samples concentration evaluated against oral bacteria were between 0.195 µg/mL to 400 µg/mL. The positive control concentrations used (CHD: chlorhexidine) ranged from 0.115 µg/mL to 400 µg/mL. DMSO at 5% concentration was used as untreated control and had no effect on the tested bacteria.

The free ligand Hatc-Ph and compounds **1** and **3** showed MIC and CBM values over 100 or 400 µg/mL for all assayed microorganisms, classifying them as not active against those bacteria. Hatc-Me presented considerable MIC and MBC values (<12.5 µg/mL) in three tested strains, while its cobalt(III) complex (**1**) derivate has not shown relevant biological activity (MIC and MCB = 400 µg/mL). Surprisingly, the phenyl containing ligand Hatc-Ph did not present activity, but complex **2** showed promising activity against all the investigated bacteria, especially for *S. sanguinis* strain in which the MIC and MCB are equal to 0.39 µg/mL, showing bactericidal and bacteriostatic effects, and, in this case improving the activity by complexation, as previously observed [3,13,27]. Neutral nickel [37] and manganese [13] complexes of the type [M(atc-R)_2_] (R = Me, Ph) reported previously presented similar MIC values for *Mycobacterium tuberculosis*, (MIC of [M(atc-Ph)_2_] was 0.78 µg/mL for M = Mn and 0.95 µg/mL for M = Ni) as the analogous cobalt complex [Co(atc-Ph)_2_]^+^ (**2**) presented in this work, but not showing the same trend in the biological activity with the change in the substituting R group.

**Figure 3:**
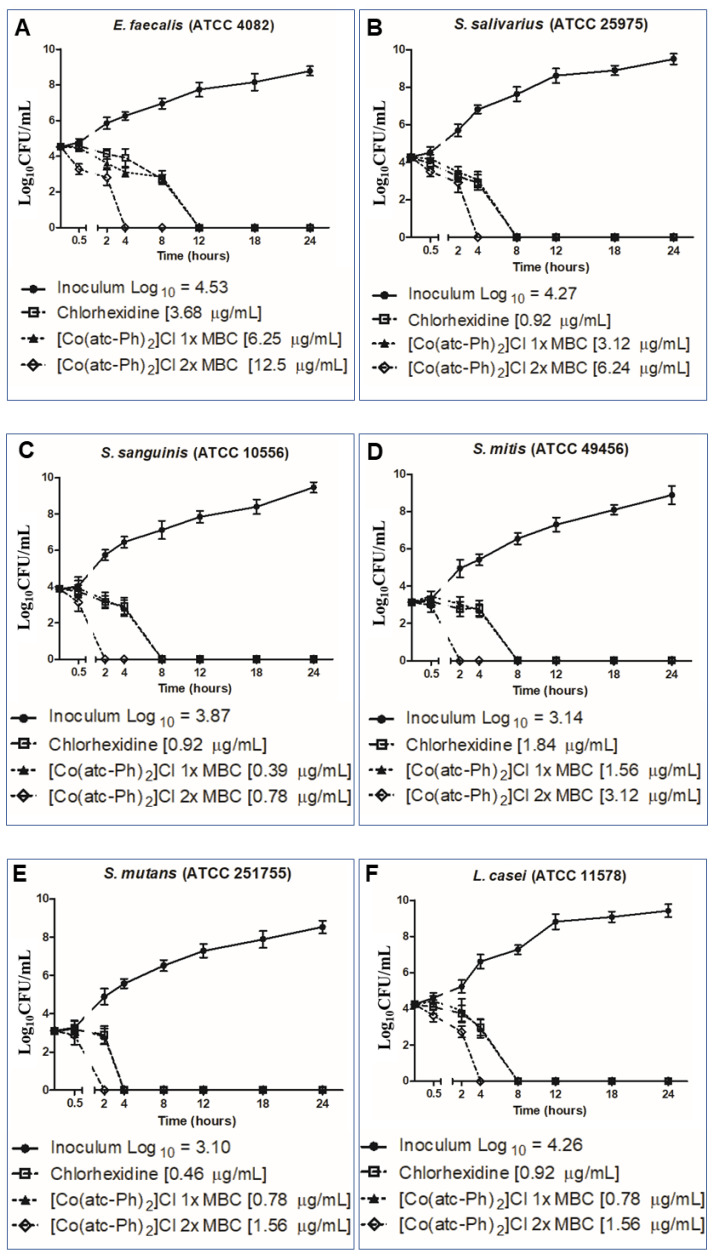
Time–kill curve profiles for complex **2** for caries bacteria as an antibacterial agent in different concentrations of MBC results, (**A**) *E. faecalis*; (**B**) *S. salivarius*; (**C**) *S. sanguinis*; (**D**) *S. mitis*; (**E**) *S. mutans*; (**F**) *L. paracasei*.

To investigate even further the antimicrobial potential of the complexes, time–kill curve assays were performed against six bacteria strains of complex **2**, the most active compound. The curves were drawn using minimal bactericidal concentration (MBC) results obtained for complex **2** and are shown in Figure 3. Complex **2** had bactericidal action for all strains evaluated in less than 24 h, in the two concentrations evaluated. The compound at one time the MBC killed *Streptococcus mutans* in 4 h; *S. salivarius*, *S. sanguinis*, *S. mitis* and *Lactobacillus paracasei* in 8 h; and *Enterococcus faecalis* in 12 h. The compound at two times the MBC concentration killed all colonies of *S. sanguinis* and *S. mutans* in 2 h; *E. faecalis*, *S. salivarius*, *S. sanguinis* and *S. mitis* in 4 h; and *L. paracasei* in 8 h. The chlorhexidine used as positive control also presented the same bactericidal effected in less than 24 h, but the only strain that the antimicrobial eliminated faster than the compound was *L. paracasei*, which suggests that complex **2** has antibacterial action that is as fast as chlorhexidine.

The structure–activity relation between the complexes and their antimicrobial activity is clearly affected by the peripheral group change in the thiosemicarbazone, since the antibacterial activity is dependent on the compound permeation through the cell walls of microorganisms [38] and their membrane–cell interaction. The presence of hydrophilic groups in the organic ligands, for instance, considerably reduces the biological activity as shown previously [39]. In our case, changing the CH_3_ group to the phenyl group led to an improvement in the antibacterial activity potency, which corroborates with the higher lipophilicity predicted for the phenyl-derived group.

#### 2.3.2. Antiviral Activity

**Figure 4:**
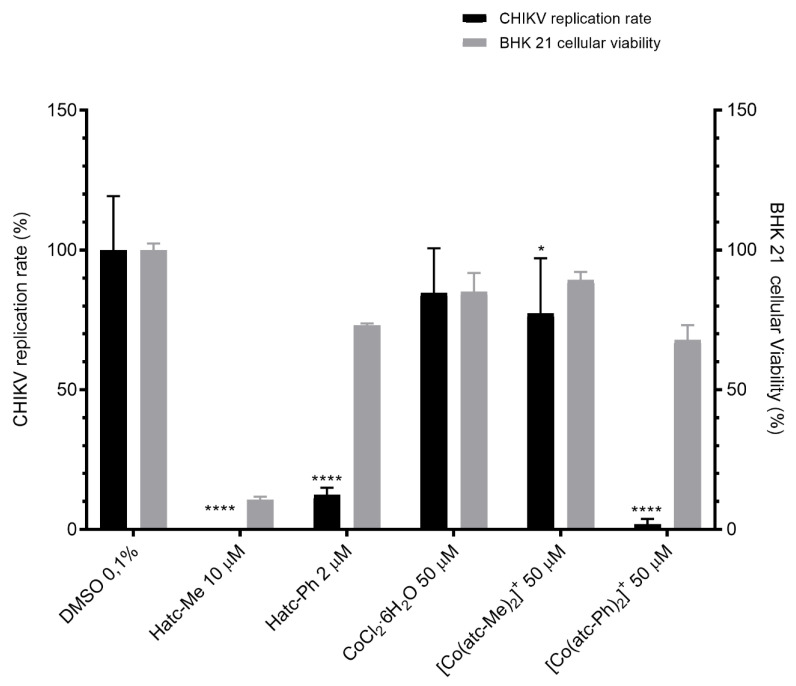
Activity of compounds on chikungunya virus (CHIKV) infection and viability of baby hamster kidney (BHK) 21 cells. BHK 21 cells were infected with CHIKV *nanoluc* and simultaneously treated with the compound. After 16 h post-infection (p.i.), cells were lysed, and a Renilla luciferase assay was performed to assess CHIKV replication rates. In parallel, a 3-(4,5-dimethylthiazol-2-yl)-2,5-diphenyl tetrazolium bromide (MTT) assay was performed. Mean values of a minimum of three independent experiments each measured in triplicate are represented. **** *p* < 0.001/* *p* < 0.05 were considered significant.

The effects of the free ligands and complexes on CHIKV infection were also studied, and the cellular viability was evaluated on baby hamster kidney (BHK) 21 cells, which were used for infection in the antiviral assays. The antiviral activity of the compounds against CHIKV was determined by using a recombinant CHIKV replicon that expresses the *nanoluciferase* reporter (CHIKV *nanoluciferase*) [40]. First, BHK 21 cells were treated with each compound at 50, 10 and 2 µM (0.1% of DMSO) diluted in cell culture media for 16 h, and cell viability was measured through a 3-(4,5-dimethylthiazol-2-yl)-2,5-diphenyl tetrazolium bromide (MTT) assay to access cytotoxicity. DMSO was used as non-treated control during the experiment. Cells tolerated each compound at different concentrations, and data of the maximum tolerated concentration are shown for each compound (Figure 4). Then, BHK 21 cells were infected with CHIKV *nanoluc* at a multiplicity of infection (MOI) of 0.01 and simultaneously treated with Hatc-Me 10 µM, Hatc-Ph 2 µM, CoCl_2_ꞏ6H_2_O 50 µM, [Co(atc-Me)_2_]Cl 50 µM (**1**) and [Co(atc-Ph)_2_]Cl 50 µM (**2**). The inhibition of the CHIKV infection in the presence of the free ligands Hatc-Me at 10 µM and Hatc-Ph 2 µM was significantly reduced to 0 and 12% (*p* < 0.01). Otherwise, the methyl-containing ligand presented low cell viability on BHK 21 cells (11%), demonstrating that the anti-CHIKV effect observed for this free ligand was a consequence of cell cytotoxicity. Alternatively, the phenyl ligand was well tolerated (73%) to BHK 21 cells, suggesting the potential antiviral property of this compound (Figure 4).

In order to better understand the structure–activity relationship, the metal precursor was also evaluated in regard to its activity on CHIKV replication. It was observed that the cobalt starting material salt at 50 µM presented low inhibition of the virus replication and had no toxicity on BHK 21 cells (Figure 4). As expected, the effect of the complexes on virus replication showed highly significant inhibition only for complex **2** compared to the untreated control. Treatment of CHIKV-infected cells with [Co(atc-Ph)_2_]Cl 50 µM reduced up to 85% of virus replication, and cell viability was not affected compared to the treatment with the Hatc-Ph ligand. Surprisingly, the results confirmed that the complexation significantly affected the activity on virus replication without increasing the toxicity. This was mainly observed when comparing the concentrations of treatment of the free ligand (2 µM) or complexed molecule (50 µM). It seems that cells tolerated higher concentrations of the Hatc-Ph when complexed to the metal. This fact suggests that the metal ions play a role in the mechanism of action of the compounds.

Taken together, these biological results suggested that the higher volume and increased lipophilicity of the substituent group on the *N*(4) position of the thiosemicarbazone moiety play a vital role in determining both antibacterial and antiviral properties, and in the present investigation, the phenyl group is most optimal. From our results, it appears that, in general, the best activity is found in compounds of higher lipophilicity. Overall, the present study identified cobalt(III) complex **2** as a potential antiviral against CHIKV replication.

### 2.4. Molecular Docking

Antibacterial activity of the most active complex was also theoretically investigated and compared with that of CHD (positive control) to identify the interactions by binding sites on the structure of *S. mutans*, *S. mitis*, *S. sanguinis*, *L. paracasei* and *E. faecalis* bacteria. For the validation of the protocols performed in molecular docking, redocking of the cocrystal of ligands was performed using AutoDock4 program to validate structure–activity relationships.

The comparison between antibacterial activity and those calculated theoretically for the [Co(L^Ph^)_2_]^+^ complex and CHD are in agreement for all bacteria, except for *S. sanguinis*. In the docking, the complex showed better results (high binding affinity) with *S. mutans* and *L. paracasei* bacteria when compared to CHD, while CHD presented better interaction with *S. mitis*, *S. sanguinis* and *E. faecalis* bacteria strains. Thus, the observed trend in the binding free energy was found to be consistent with the experimental inhibition trend for the [Co(atc-Ph)_2_]^+^ complex and CHD. The obtained ΔG values and estimated inhibition constant (Ki) of the [Co(atc-Ph)_2_]^+^ and CHD with *S. mutans*, *S. mitis*, *S. sanguinis*, *L. paracasei* and *E. faecalis* bacteria are shown in Table S3.

**Figure 5:**
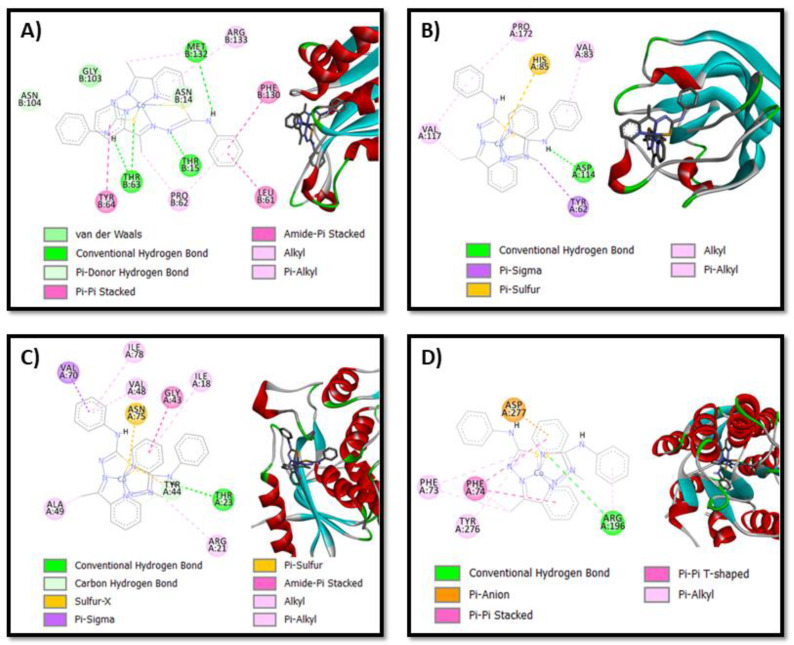
Interaction network between the [Co(atc-Ph)_2_]^+^ complex and (**A**) *S. mutans*; (**B**) *S. mitis*; (**C**) *S. sanguinis*; and (**D**) *L. paracasei* protein structures. For clarity, the non-polar hydrogen atoms of the metal complex are omitted.

In Figure 5 and Figures S6–S11, the images of the interaction conformations of the [Co(atc-Ph)_2_]^+^ complex and CHD are shown. It is evident from the results obtained that both the [Co(atc-Ph)_2_]^+^ complex and CHD can successfully bind to one or more amino acids of all bacteria. The results of ΔG obtained were highly exothermic, which reveals good orientation and proximity, demonstrating the increment of the binding affinity [41]. The free binding energy between the [Co(atc-Ph)_2_]^+^ complex and bacteria was found to be −9.55 kcal/mol for *L. paracasei*, followed by −8.66 kcal/mol found for *S. sanguinis*. The greatest energy value was found for the *S. mitis* protein structure (−3.45 kcal/mol). On the other hand, the best free binding energy between CHD and bacteria was found for *E. faecalis* (−11.24 kcal/mol), followed by *S. sanguinis* (−9.49 kcal/mol), and the highest value was found for *S. mitis* (−3.03 kcal/mol). Energy derived from the cobalt complex interaction with the *L. paracasei* structure was lower compared to the energy derived from CHD interaction with the same bacteria structure, suggesting that the complex couple is more stable, a fact observed for other metal complexes in the literature [41,42]. This observation is also in accordance with the antibacterial assay results which show a higher biological activity for [Co(atc-Ph)_2_]^+^ (MIC/MBC = 0.39/0.39 µg/mL) than for CHD (MIC/MBC = 0.92/0.92 µg/mL). In all the investigated systems, predominantly hydrogen bonds were observed, and in almost all cases, aromatic–aromatic, aromatic–alkyl or aromatic-sulfur interactions were found.

The docked pose between the [Co(L^Ph^)_2_]^+^ complex and *L. paracasei* (Figure 5D) shows a considerably strong H bond (3.18 Å in length) involving the N-H group from thiosemicarbazone ligand and a sulfur atom of amino acid Arg 196. Additionally, two pi–pi interactions between the pyridinic rings of both ligands in the complex and the Ph group of amino acid Phe 74, which are 4.74 Å and 4.78 Å in length, respectively, were observed. Similarly, the [Co(atc-Ph)_2_]^+^ complex and *S. sanguinis* also have a hydrogen bond between the N-H group of the complex and sulfur atom of amino acid Thr 63 with a length of 3.76 Å. Furthermore, two shorts H bonds between the N-H group of the complex and an oxygen atom of amino acids Thr 63 (1.81 Å) and Met 132 (2.35 Å) are observed. These short H bonds may justify the fact that the antibacterial activity of *S. sanguinis* had the lowest MIC and CBM value among all the tested bacteria. More details about the calculated H bond and pi–pi and pi–S distances of intermolecular interactions of the cobalt complex with amino acid residues of all the bacteria are shown in Table S4. Finally, according to all these presented interactions, the [Co(atc-Ph)_2_]^+^ complex can effectively bind to all bacteria and, subsequently, displays great potential as antibacterial agents.

## 3. Experiment

### 3.1. Materials

All chemicals were reagent grade and used without purification. The reagents and solvents 4-methyl-3-thiosemicarbazide, 4-phenyl-3-thiosemicarbazide, 2-acetylpyridine and analytical were obtained commercially. The compounds Hatc-Me and Hatc-Ph were synthesized as previously reported [43].

### 3.2. Preparation of the Complexes

The complexes [Co(atc-Me)_2_]Cl (**1**) and [Co(atc-Ph)_2_]Cl (**2**) were synthesized by slight modifications of the reported procedures described by Oliveira et al. [27] An amount of 0.25 mmol of CoCl_2_·6H_2_O was added to 0.5 mmol of Hatc-R in 10 mL ethanol. A brown solid was formed, separated by filtration and washed with *n*-hexane. The purity of the solids was confirmed by ^1^H, ^13^C NMR, LC–MS/MS and conductivity measurements.

R = Me (**1**). Yield 97.0 mg (60%). ^1^H NMR (400 MHz, DMSO-*d*_6_, δ in ppm): 2.85 (s, 9H, 2CH_3_C=N and 1CH_3_NH), 2.95 (s, 3H, CH_3_NH), 7.44 (s, 2H, Py-H), 7.85 (d, *J* = 4 Hz, 2H, Py-H), 8.03–8.09 (m, 4H, Py-H), 8.15 (s, 1H, NHCH_3_), 8.79 (s, 1H, NHCH_3_). ^13^C NMR (101 MHz, DMSO-*d*_6_, δ in ppm): 15.0 (CH_3_), 32.1 (CH_3_), 124.6 (CH), 126.6 (CH), 139.9 (CH), 148.7 (CH), 155.4 (CH), 157.2 (C), 178.2 (C). (+)-HRESIMS *m*/*z* 473.0734 [M]^+^ (calcd for C_18_H_22_ClCoN_8_S_2_, 473.0735 (Δ −0.21 ppm)). HPLC: *t*_R_ = 4.8 min.

R = Ph (2). Yield 107.0 mg (68%). ^1^H RMN (400, DMSO-*d*_6_, δ in ppm): 3.00 (s, CH_3_, 6H), 7.08 (t, *J* = 8 Hz, Ph, 2H), 7.38 (t, *J* = 8 Hz, Ph, 4H), 7.52 (dd, *J* = 7 Hz, *J* = 2 Hz, Py, 2H), 7.76 (dd, *J* = 8 Hz, Ph, 4H), 8.02 (d, *J* = 8 Hz, Py, 2H), 8.14–8.19 (m, Py, 4H), 10.42 (s, NH, 2H). ^13^C NMR (101 MHz, DMSO-*d*_6_, δ in ppm): 15.8 (CH_3_), 120.4 (CH), 123.3 (CH), 125.5 (CH), 127.4 (CH), 128.7 (CH), 139.9 (CH), 140.1 (C), 149.2 (CH), 156.7 (CH), 158.7 (C), 175. (C). (+)-HRESIMS *m*/*z* 597.1049 [M]^+^ (calcd for C_28_H_26_ClCoN_8_S_2_, 597.1048 (Δ 0.17 ppm)). HPLC: *t*_R_ = 7.7 min.

### 3.3. Instruments

Nuclear magnetic resonance (NMR) experiments were accomplished with a Bruker Ascend^TM^ 400 Avance III HD spectrometer (9.2 T) at 400 MHz (^1^H) and 100 MHz (^13^C) at 25 °C and referenced using Tetramethylsilane (TMS)(δ_TMS_ = 0.00) as internal standard or residual solvent signals of dimethylsulfoxide-*d*_6_ (DMSO-*d*_6_) at δ 2.50 and 39.5 ppm, respectively, for ^1^H and ^13^C. The molar conductivities of the complexes were measured using an Orion Star Series conductometer (1 × 10^−3^ mol L^–1^ MeOH solutions). High-resolution electrospray ionization mass spectra (HRESIMS) were measured on a Bruker Daltonics (microTOF-Q II) spectrometer, operating in the positive mode. Methanol–water (7:3, *v*/*v*) was used as solvent system, and the samples were infused into the Electrospray Ionization (ESI) source at a flow rate of 180 µL/h. N_2_ was employed as drying gas at a flow rate of 4 L/min and as nebulizer gas at 130 psi. The temperature of the nebulizer was set at 180 °C, and a potential of 3.5 kV was applied in the capillary. The collision energies were adjusted according to the precursor ion (charged complexes [M]^+^). The calculated values for the charged complex ions were made using ChemDraw Ultra 14.0. LC–MS data were recorded on Agilent mode Infinity 1260 coupled with a Q-TOF spectrometer of the Agilent^®^ model 6520 B with an electrospray ionization source (IES) at the Nanobiotechnology Laboratory of the Federal University of Uberlândia (IBTC-UFU). The HPLC column was an Agilent model Poroshell, 100 × 3 mm, with a pore size of 2.7 µm. The mobile phase consisted of (A) deionized water 0.1% formic acid and (B) methanol. The following solvent gradient was 10% of B (0 min), 98% of B (0–10 min), and 100% of B (10–17 min). The ionization parameters were nebulizer pressure of 20 psi, drying gas at a flow rate of 8 L/min at a temperature of 220 °C and a capillary voltage of 4.5 kV.

### 3.4. Antibacterial Assays

#### 3.4.1. Determination of MICs and MBC

The minimum inhibitory concentration (MIC) and the minimum bactericidal concentration (MBC) of the compounds were determined in triplicate. For the MIC, the microdilution broth method in 96-well microplates was utilized. Standard strains acquired from the American Type Culture Collection were used: *Streptococcus mutans* (ATCC 251755), *S. mitis* (ATCC 49456), *S. sanguinis* (ATCC 10556), *S. sobrinus* (ATCC 33478), *Lactobacillus paracasei* (ATCC 11578), *S. salivarius* (ATCC 25975), and *Enterococcus faecalis* (ATCC 4082). The methodology employed was in accordance with the guidelines of the Clinical and Laboratory Standards Institute, with some modifications that have been previously described in other studies by our research group [44], using brain heart infusion broth (BHI, Difco), and interpretation of the results was made using resazurin.

Solutions of the samples in concentration of 1.0 mg mL^−1^ were prepared in DMSO (Sigma-Aldrich, St. Louis, MO, USA), followed by dilution in BHI until obtaining concentrations ranging from 0.195 to 400 µg mL^−1^ with a final DMSO content of 5% (*v*/*v*), and this solution was used as a negative control. The inoculum was adjusted for each organism to yield a cell concentration of 5 × 10^6^ colony-forming unit (CFU) per mL. Chlorhexidine was added as positive control, and the concentrations ranged from 0.115 to 50 µg/mL. The microplates were incubated at 37 °C for 24 h in microaerophilic conditions. Afterwards, resazurin (30 uL) in aqueous solution (0.02% *v*/*v*) was added to the microplates to indicate bacteria viability [44]. The MIC was interpreted as the minimum concentration of the sample that can inhibit the growth of bacteria.

To determine the MBC, an aliquot (10 µL) of the inoculum from each well of the MIC’s microplates was removed prior of resazurin and seeded in brain heart infusion agar (BHIa). Afterwards, the plates were incubated at 37 °C for 24 h. At the end of the incubation, it was defined as the lowest concentration of the sample at which no bacterial growth occurred.

#### 3.4.2. Time–Kill Curves

Time–kill assays against the strains *Streptococcus mutans* (ATCC 251755), *S. mitis* (ATCC 49456), *S. sanguinis* (ATCC 10556), *Lactobacillus paracasei* (ATCC 11578), *S. salivarius* (ATCC 25975), and *Enterococcus faecalis* (ATCC 4082) were performed in triplicate, as described by D’arrigo et al. (2010) [45], with some modifications. The 96-well microplates containing the most promising compound at concentrations of one and two times the MBC value for the respective strains were inoculated with the target microorganism at an initial bacterial density of 5 × 10^6^ CFU/mL and incubated in microaerophilic conditions. To count viable colonies, aliquots were removed at 0 min and 30 min and at 2, 4, 8, 12, 18 and 24 h. The diluted samples (50 µL) were spread onto BHI agar plates, incubated at 37 °C under appropriate atmosphere, and counted after the growth period. Time–kill curves were constructed by plotting log_10_ CFU/mL vs. time on the GraphPad Prism (version 8.0) software. Chlorhexidine and bacteria suspension without added compounds were used as the positive and negative control, respectively.

### 3.5. Antiviral Assays

#### 3.5.1. Cell Culture

Baby hamster kidney cells (BHK 21, ATCC^®^ CCL-10™), which are susceptible to CHIKV infection [46], were maintained in Dulbecco’s modified Eagle’s medium (DMEM; Sigma-Aldrich) supplemented with 100 U/mL penicillin (Hyclone Laboratories, Logan, UT, USA), 100 mg/mL streptomycin (Hyclone Laboratories, USA), 1% non-essential amino acids (Hyclone Laboratories, USA) and 1% fetal bovine serum (FBS, Hyclone Laboratories, USA) in a humidified 5% CO_2_ incubator at 37 °C.

#### 3.5.2. In Vitro Cytotoxic Activity Evaluation by MTT Assays

Stock solutions of the free ligands and cobalt complexes were firstly prepared in DMSO at 50, 10 and 2 mM, following dilutions in cell culture media to achieve a concentration in micromolar and DMSO at 0.1%. Dimethyl sulfoxide (DMSO) was used as a non-treated control. Cell viability was measured through MTT (3-(4,5-dimethylthiazol-2-yl)-2,5-diphenyl tetrazolium bromide) (Sigma-Aldrich) assay. BHK 21 cells were cultured in 96-well plates and treated with 50, 10 and 2 µM of each compound for 16 h at 37 °C with 5% of CO_2_. After treatment, compound-containing media was removed from 96-well plates, and MTT at 1 mg/mL solution was added to each well, incubated for 1 h and replaced with 100 μL of DMSO to solubilize the formazan crystals. The absorbance was measured at 560 nm on a Glomax microplate reader (Promega). Cell viability was calculated according to the equation (T/C) × 100%, in which T and C represent the optical density of the treated well and control groups, respectively. The highest non-cytotoxic concentration, ≥80%, was selected for further analyses in the antiviral assay.

#### 3.5.3. Assessment of CHIKV Replication

To analyze the antiviral activity of each compound, BHK 21 cells were seeded at a density of 5 × 10^4^ cells per well into 48-well plates 24 h prior to infection. Then, CHIKV nanoluciferase at a multiplicity of infection (MOI) of 0.01 and compounds, at the highest non-cytotoxic concentration selected in the MTT assay, were simultaneously added to cells in cell culture media. Samples were harvested in Renilla luciferase lysis buffer (Promega) at 16 h post-infection (h.p.i.), and virus replication was quantified by measuring nanoluciferase activity using the Renilla luciferase Assay System (Promega). DMSO 0.1% was used as non-treated control.

### 3.6. Statistical Analysis

Individual experiments were performed in triplicate, and all assays were performed a minimum of three times in order to confirm the reproducibility of the results. Differences between means of readings were compared using analysis of variance (one-way or two-way ANOVA) or Student’s *t*-test using Graph Pad Prism 8.0 software (Graph Pad Software). *p* values ≤ than 0.01 were considered statistically significant.

### 3.7. Molecular Docking Study

Molecular docking studies were performed using autodock4.2 (AD4) [47] with the [Co(atc-Ph)_2_]^+^ complex and CHD (positive control). The crystal structures of the complexes [Co(atc-Ph)_2_]Cl (CCDC 1416048) and CHD (CCDC 988972) were obtained from The Cambridge Crystallographic Data Centre, and the crystal structures of *S. mutans* (PDB 5C2O), *S. mitis* (PDB 3LE0), *S. sanguinis* (PDB 4N82), *L. paracasei* (PDB 6CHK) and *E. faecalis* (PDB 4LRL) bacteria were obtained from Protein Data Bank. No structures were found for *S. sobrinus* and *S. salivarius bacteria*. The re-dock procedures were conducted using the AutoDockTools 1.5.6 graphical interface along with autogrid4 and AD4. To prepare the cobalt complex and bacteria structures for molecular docking, AutoDockTools 1.5.6 was added to Gasteiger charges [48] and converted to the crystal structures required for AD4. The Lamarckian genetic algorithm in AutoDock was as follows: population (150 individuals); maximum energy evaluations (25,000,000); maximum generations (27,000); one individual surviving into next generation; genetic algorithm docking runs (150). A ranked cluster analysis was performed using a root mean square (RMS) tolerance of 2.0 Å on each docking calculation. The grid map and centers used can be seen in Table S2. The interaction pictures were generated using the Discovery Studio Visualizer program.

## 4. Conclusions

Screening of two cobalt(III) complexes in seven bacteria strains and on the chikungunya virus revealed a remarkably high potency for the phenyl group (**2**) compared to the methyl-derived group (**1**). The coordination of Hatc-Ph to the Co(III) was able to improve the antibacterial and antiviral activities when compared with the free ligand without an increase in the cytotoxicity, as verified by the test on healthy cells. Furthermore, the biological studies showed that small structural changes in the peripheral group of the thiosemicarbazone can substantially affect the antibacterial and antiviral activities. The results of biological activity have shown to be dependent on the metal ion and the phenyl group. The antiviral assays revealed a remarkable level of activity of free ligands Hatc-Me and Hatc-Ph. The coordination of the ligands to the metal ion Co(III) led to a substantial increment in the antiviral activity and low cytotoxicity on healthy cells, especially for complex **2**. Additionally, the data of both antibacterial and antiviral assays successfully established a structure–activity relationship, in which the lipophilic characteristic was important for both biological assays. Based on this study, complex **2** was identified as a potential antibacterial and antiviral agent. A molecular docking study was also carried out to explain the binding pattern of most active cobalt complex (**2**) toward *S. mutans*, *S. mitis*, *S. sanguinis*, *L. paracasei*, and *E. faecalis* bacteria strains. This study revealed that complex **2** shows the best affinity towards two bacteria, predominantly *L. paracasei* and *S. sanguinis*. This may be explained by the enhanced lipophilic property of the central metal ion. The results presented here could provide useful insights for future practical applications of cobalt(III) complexes as antiviral and antibacterial agents. Furthermore, studies are underway in our laboratories to evaluate the promising compound in order to investigate the real participation of cobalt in the biological activity.

## Figures and Tables

**Table 1:** Minimum inhibitory concentration (MIC, µg/mL) and minimum bactericidal concentration (MBC, µg/mL) in µg/mL against buccal bacteria strains.

Bacterial Strains	Hatc-Me		Hatc-Ph		[Co(atc-Me)_2_]^+^		[Co(atc-Ph)_2_]^+^		CoCl_2_·6H_2_O		CHD *	
	MIC	MBC	MIC	MBC	MIC	MBC	MIC	MBC	MIC	MBC	MIC	MBC
*Streptococcus mutans* (ATCC 251755)	12.5	12.5	100	100	400	400	0.78	0.78	100	100	0.46	0.46
*Streptococcus mitis* (ATCC 49456)	12.5	12.5	100	100	400	400	1.56	1.56	100	100	1.84	1.84
*Streptococcus sanguinis* (ATCC 10556)	6.25	6.25	100	100	400	400	0.39	0.39	100	100	0.92	0.92
*Streptococcus sobrinus* (ATCC 33478)	25	25	400	400	400	400	12.5	25	>400	>400	0.46	0.46
*Lactobacillus paracasei* (ATCC 11578)	50	50	400	400	400	400	0.78	0.78	>400	>400	0.92	0.92
*Streptococcus salivarius* (ATCC 25975)	25	25	100	100	400	400	3.12	3.12	100	100	0.92	0.92
*Enterococcus faecalis* (ATCC 4082)	>400	>400	>400	>400	400	400	6.25	6.25	>400	>400	3.68	3.68

* CHD: Chlorhexidine, positive control.

## Supplementary Materials

Supplementary Materials can be found at https://www.mdpi.com/1422-0067/21/21/8355/s1.

## Abbreviations

MIC	Minimum inhibitory concentration
CBM	Minimum bactericidal concentration
PDB	Protein Data Bank
CCDC	Cambridge Crystallographic Data Centre
AD4	Autodock4.2
RMS	Root mean square
ESI	Electrospray Ionization
TMS	Tetamethylsilane
