# Supplementary material for: Fragmentation Study, Dual Anti-Bactericidal and Anti-Viral Effects and Molecular Docking of Cobalt(III) Complexes
Source: Int J Mol Sci. 2020 Nov 7;21(21):8355. doi: 10.3390/ijms21218355 (PMC7664407; doi:10.3390/ijms21218355)
Supplement: Supplementary file 1 [file ijms-21-08355-s001.pdf]

Electronic Supplementary Information

Fragmentation Study, Dual Anti-bactericidal and Anti-viral effects and Molecular Docking of Cobalt(III) Complexes

Laísa de P. Fernandes[1], Júlia M. B. Silva[2], Daniel O. S. Martins[2], Mariana B. Santiago[2], Carlos H. G. Martins[2], Ana C. G. Jardim[2], Guedmiller S. Oliveira[3], Marcos Pivatto[1], Rafael A. C. Souza[1], Eduardo de F. Franca[3], Victor M. Deflon[4], Antonio E. H. Machado[5,6], Carolina G. Oliveira[1]*

[1] *Instituto de Química, Universidade Federal de Uberlândia, 38400-902, Uberlândia, MG, Brasil.*

[2] *Instituto de Ciências Biomédicas, Universidade Federal de Uberlândia, 38408-100, Uberlândia, MG, Brasil.*

[3] *Laboratório de Cristalografia e Química Computacional, Instituto de Química, Universidade Federal de Uberlândia, UFU, Uberlândia, MG, Brazil*

4 Instituto de Química de São Carlos, Universidade de São Paulo, 13566-590 São Carlos, SP, Brasil

[5] *Laboratório de Fotoquímica e Ciências dos Materiais, Instituto de Química, Universidade Federal de Uberlândia, 38400-902, Uberlândia, MG, Brasil.*

[6] *Unidade Acadêmica Especial de Física, Programa de Pós-Graduação em Ciências Exatas e Tecnol. Universidade Federal de Catalão, 75705-220 Catalão, GO, Brasil.*

*Corresponding author: carolina@ufu.br

Supporting Information

Figures S1 – S12	Pages 2 – 10
Tables S1– S4	Pages 11 – 12

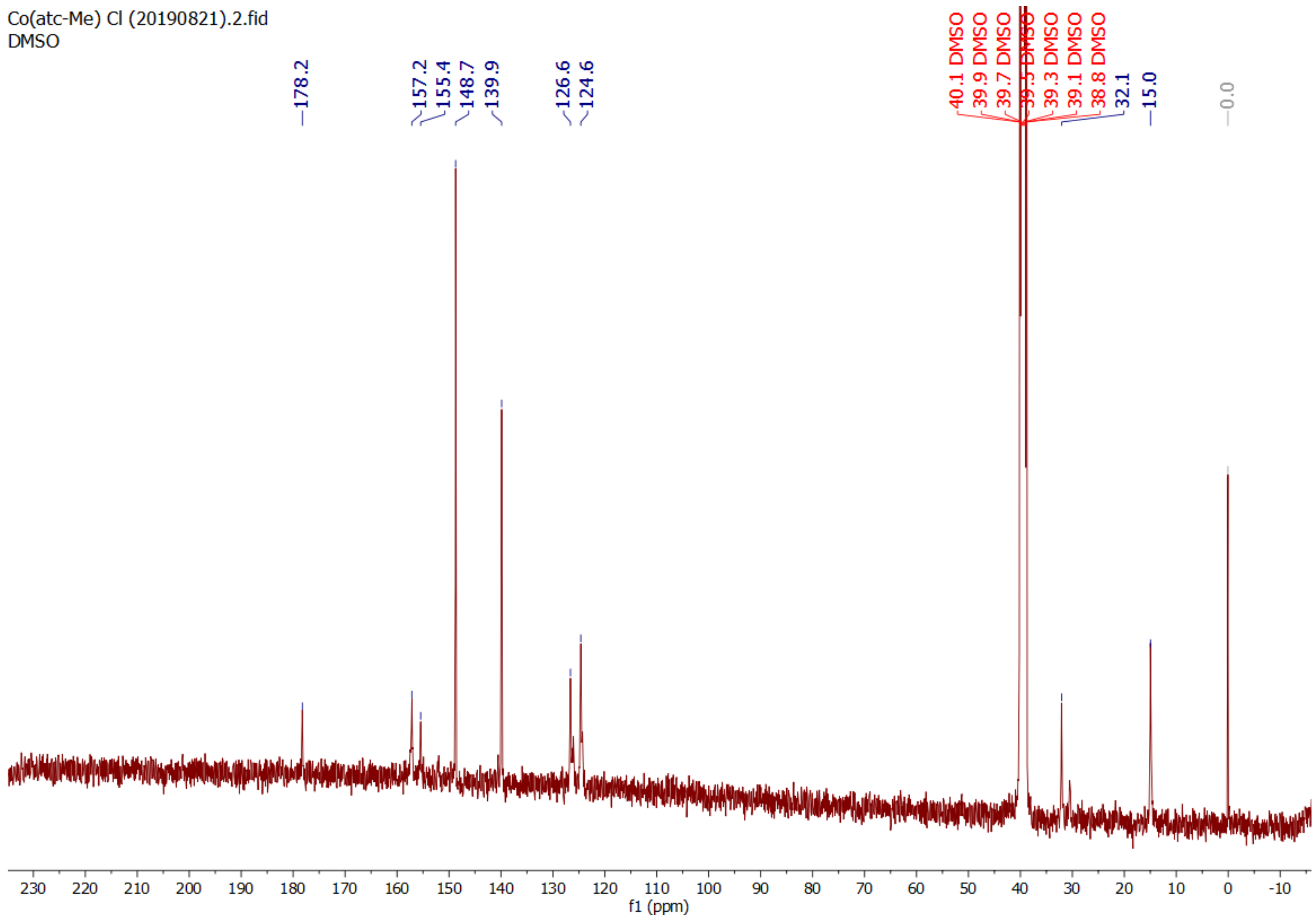

Figure S1. ^{13}C NMR spectrum of $[Co(atc\text{-}Me)_2]Cl$ in DMSO-d_6. ^{13}C NMR (δ_C): 15.0 (CH_3), 32.1 (CH_3), 124.6 (CH), 126.6 (CH), 139.9 (CH), 148.7 (CH), 155.4 (C), 157.2 (C), 178.2 (C).

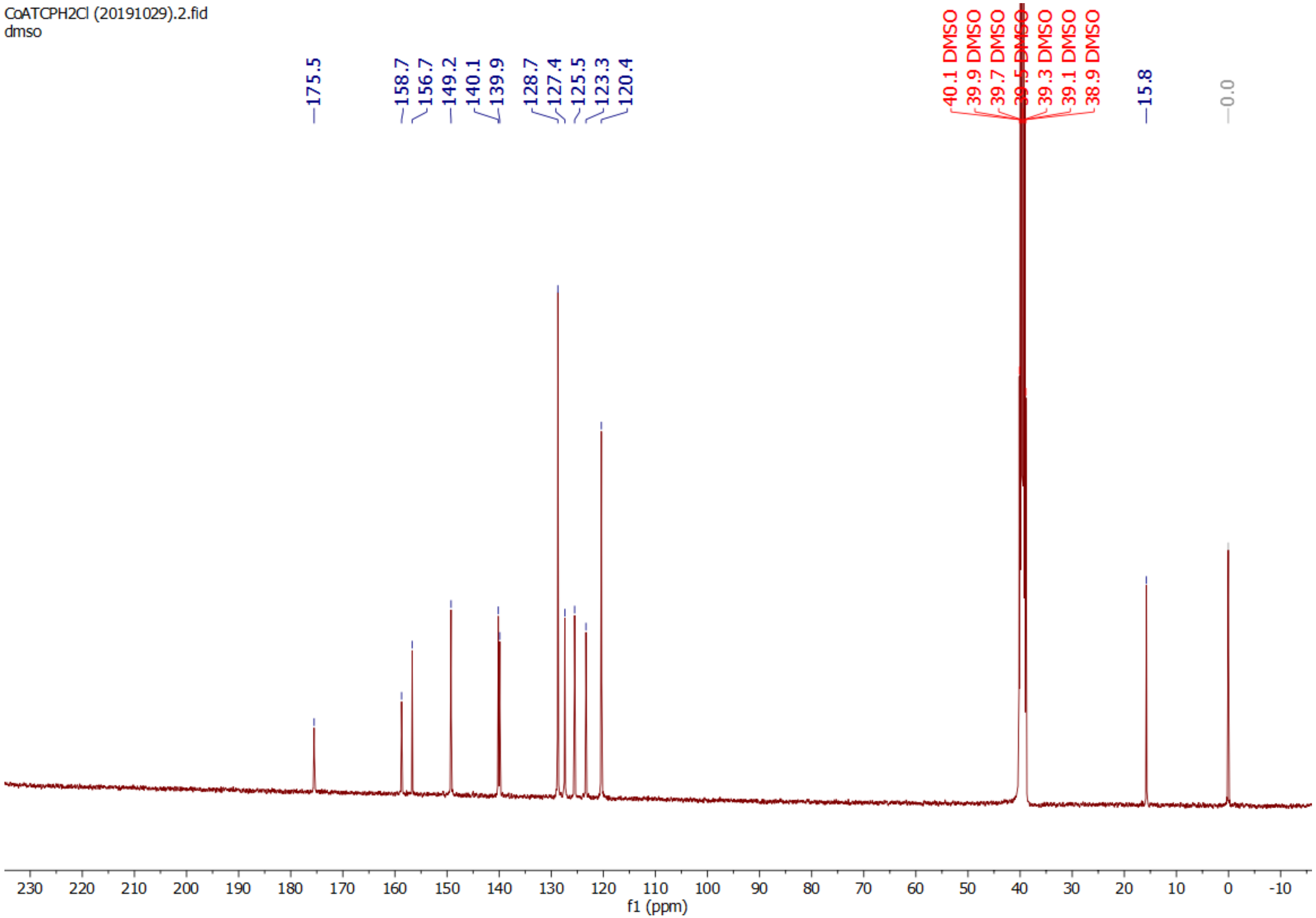

Figure S2. ^{13}C NMR spectrum of $[Co(atc\text{-}Ph)_2]Cl$ in DMSO-d_6. ^{13}C NMR (δ_C): 15.8 (CH_3), 120.4 (CH), 123.3 (CH), 125.5 (CH), 127.4 (CH), 128.7 (CH), 139.9 (CH), 140.1 (CH), 149.2 (CH), 156.7 (C), 158.7 (C), 175.5 (C).

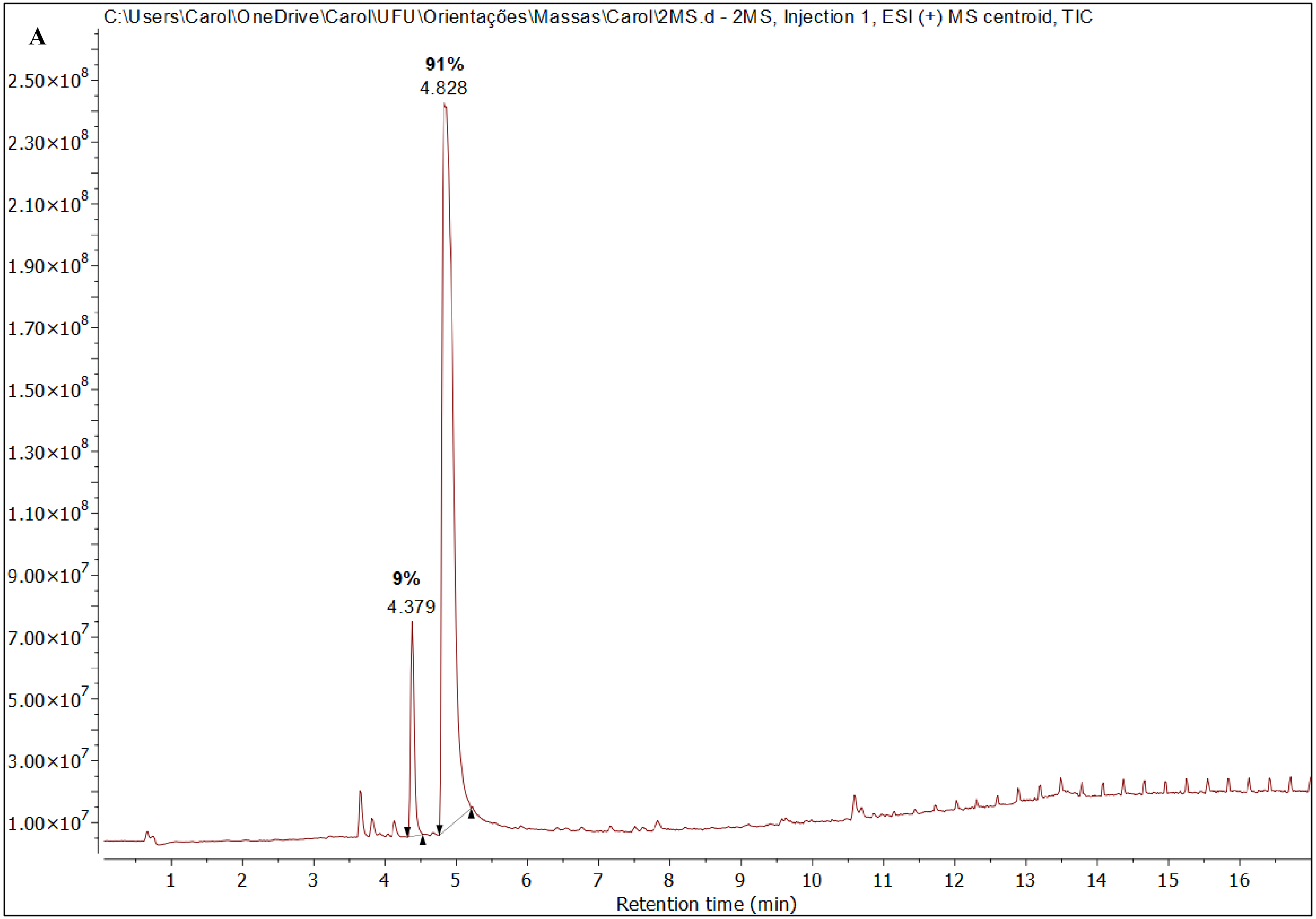

A
C:\Users\Carol\OneDrive\Carol\UFU\Orientações\Massas\Carol\2MS.d - 2MS, Injection 1, ESI (+) MS centroid, TIC
91%
4.828
9%
4.379
2.50×10^8
2.30×10^8
2.10×10^8
1.90×10^8
1.70×10^8
1.50×10^8
1.30×10^8
1.10×10^8
9.00×10^7
7.00×10^7
5.00×10^7
3.00×10^7
1.00×10^7
1 2 3 4 5 6 7 8 9 10 11 12 13 14 15 16
Retention time (min)

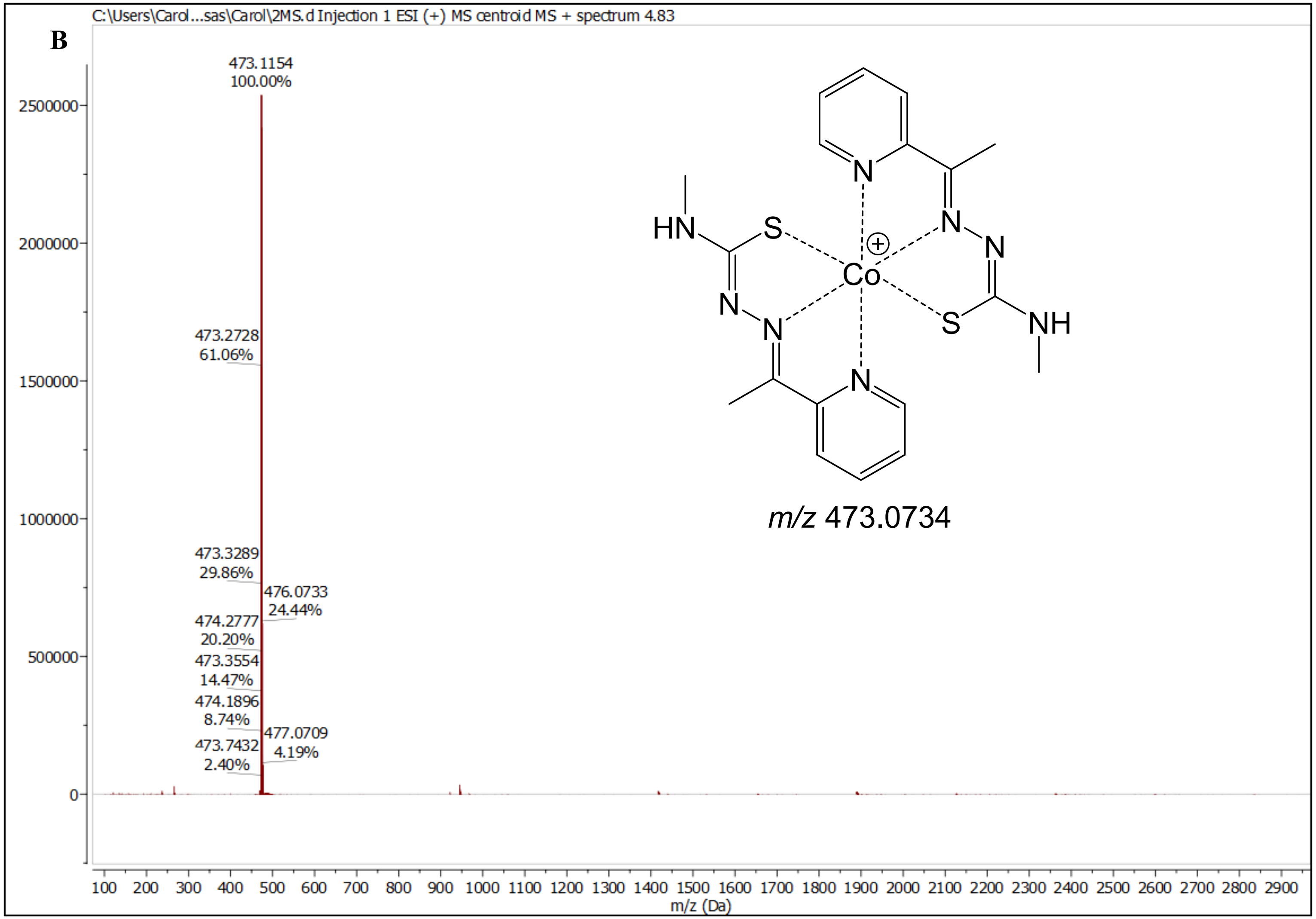

Figure S3. **A**) LC-MS chromatogram of complex **1**. **B**) Mass spectrum of complex **1** (t_R = 4.8 min) on positive mode.

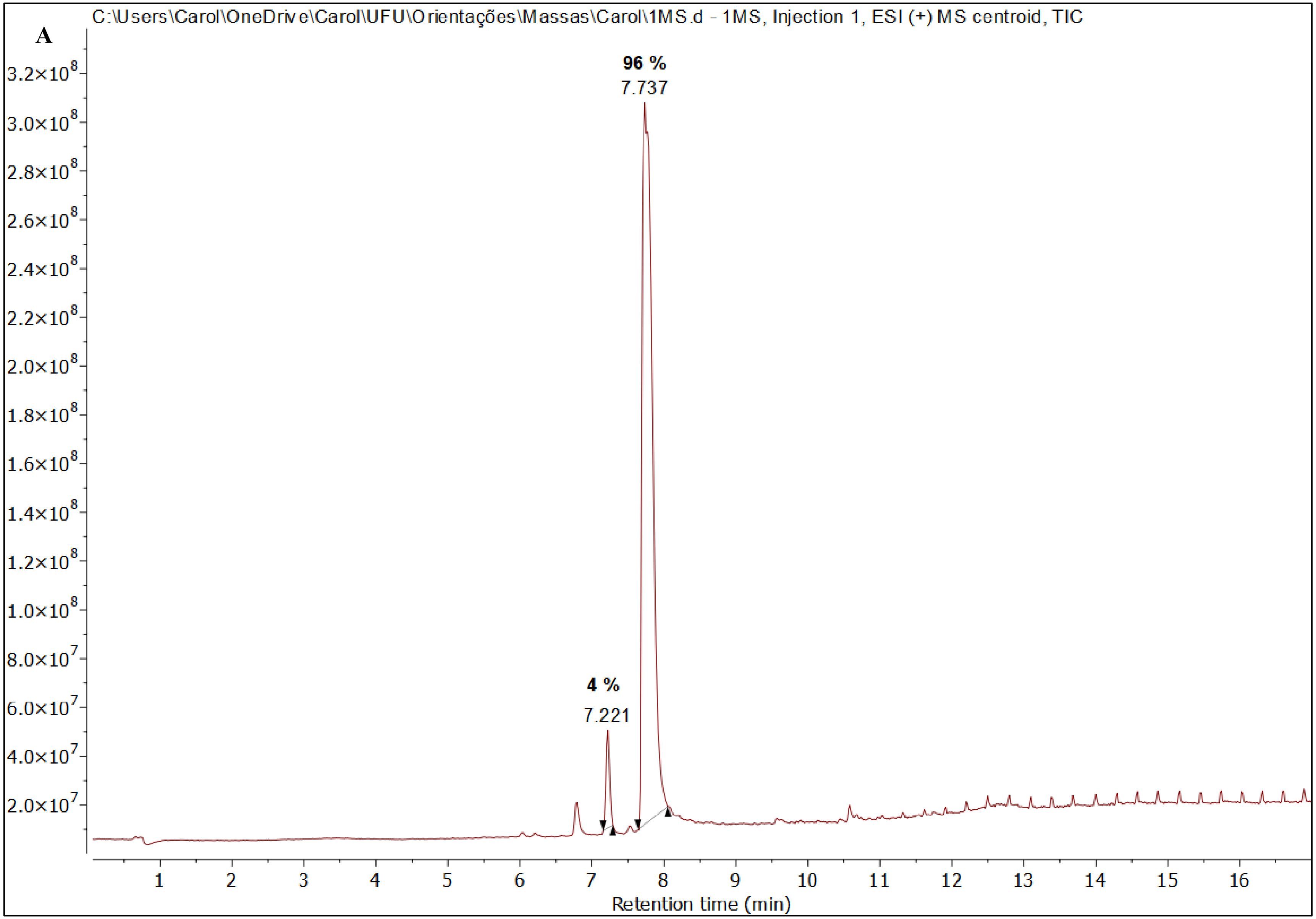
C:\Users\Carol\OneDrive\Carol\UFU\Orientações\Massas\Carol\1MS.d - 1MS, Injection 1, ESI (+) MS centroid, TIC
A
96 %
7.737
4 %
7.221
3.2×10^8
3.0×10^8
2.8×10^8
2.6×10^8
2.4×10^8
2.2×10^8
2.0×10^8
1.8×10^8
1.6×10^8
1.4×10^8
1.2×10^8
1.0×10^8
8.0×10^7
6.0×10^7
4.0×10^7
2.0×10^7
1 2 3 4 5 6 7 8 9 10 11 12 13 14 15 16
Retention time (min)

S6

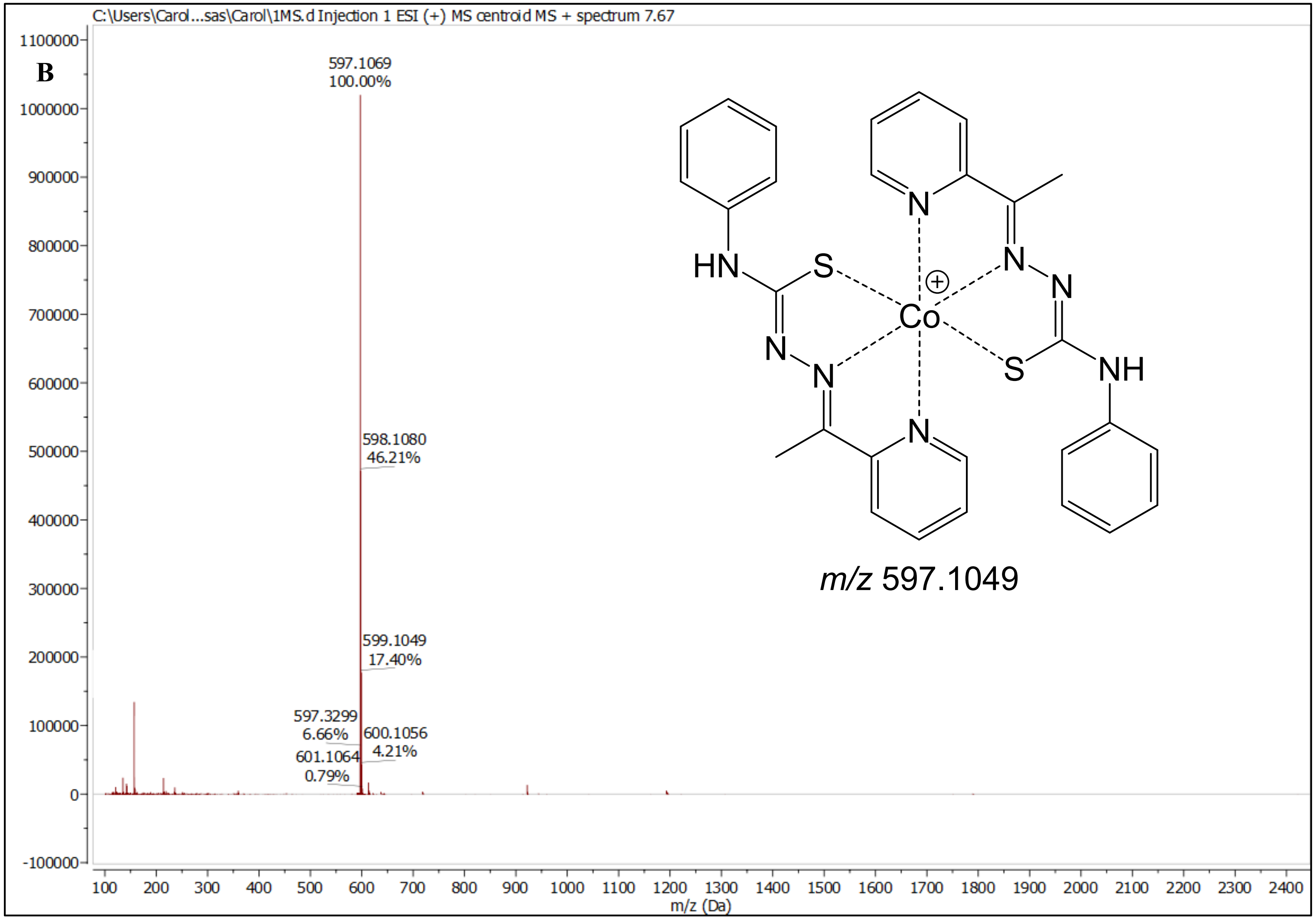

Figure S4. **A**) LC-MS chromatogram of complex **2**. **B**) Mass spectrum of complex **2** (t_R = 7.7 min) on positive mode.

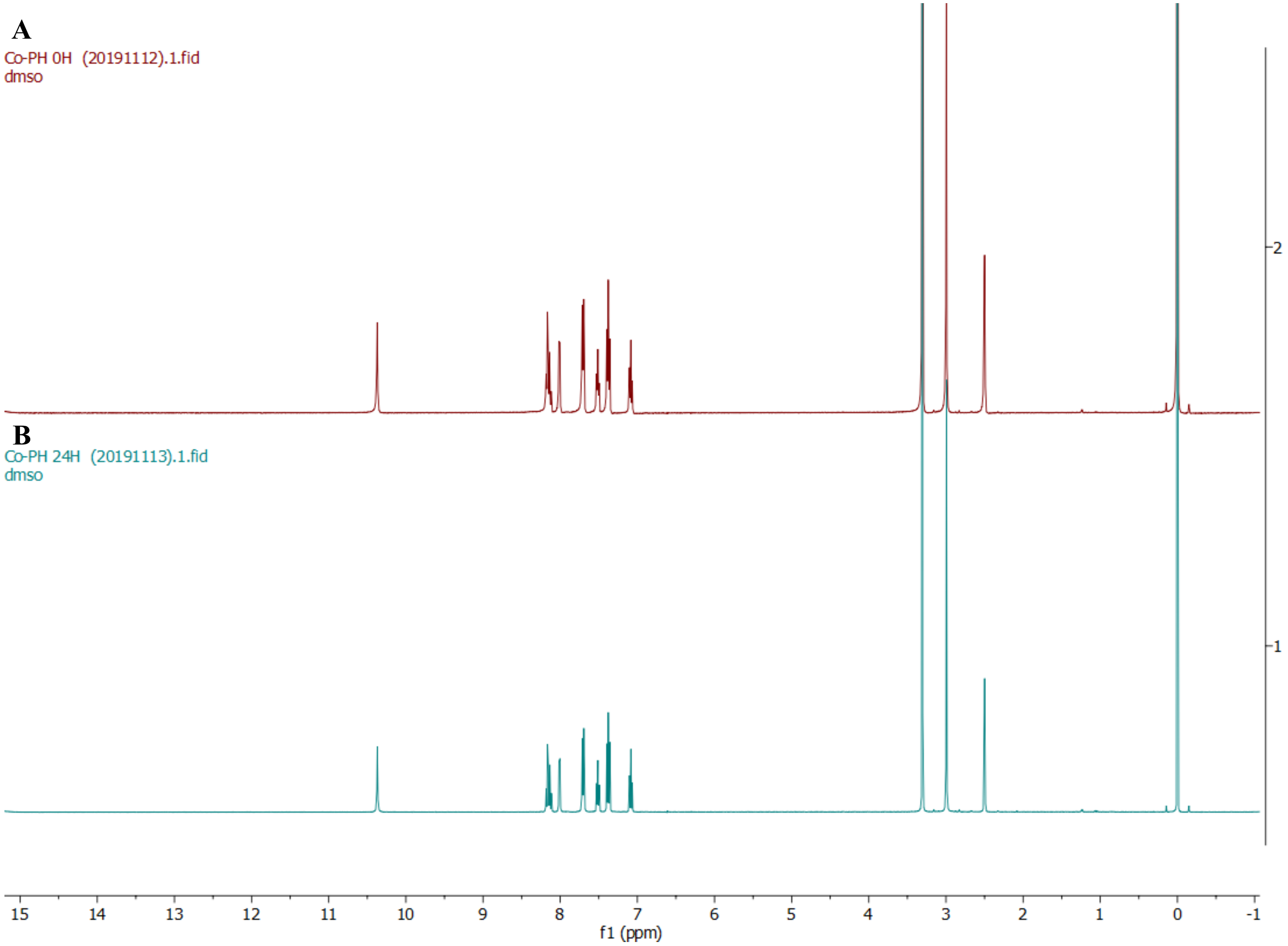

Figure S5. Stability test of **2** in DMSO-d_6 of fresh solution (**A**, 0 h) and after 24 h (**B**).

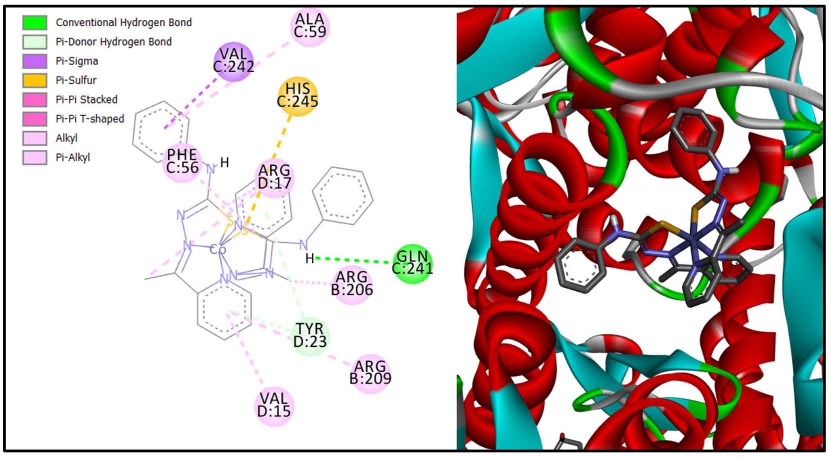

Figure S6. Interaction network between $[Co(L^{Ph})_2]^+$ complex and the *E. faecalis* protein structure. For clarity, the non-polar hydrogen atoms of the metal complex are omitted.

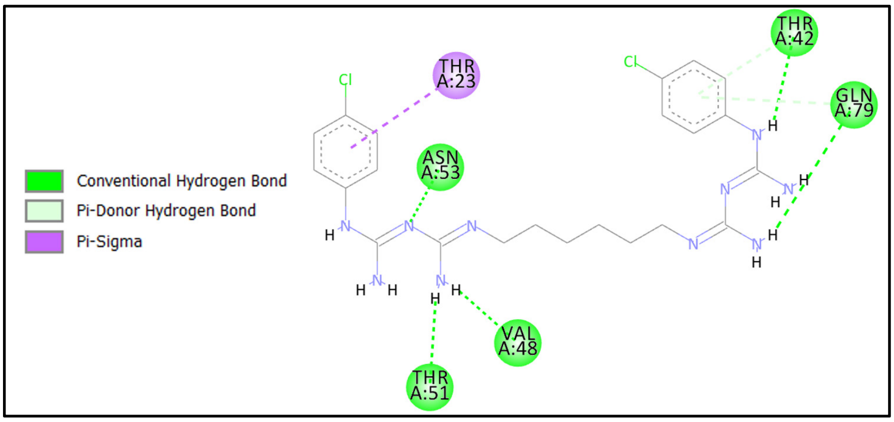

Figure S7. Interaction network between CHD and *S. mutans* protein structure. For clarity, the non-polar hydrogen atoms of CHD are omitted.

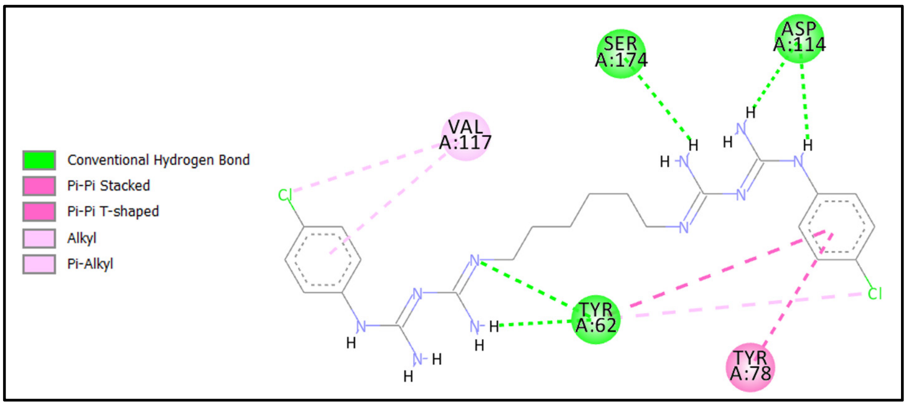

Figure S8. Interaction network between CHD and *S. mitis* protein structure. For clarity, the non-polar hydrogen atoms of CHD are omitted.

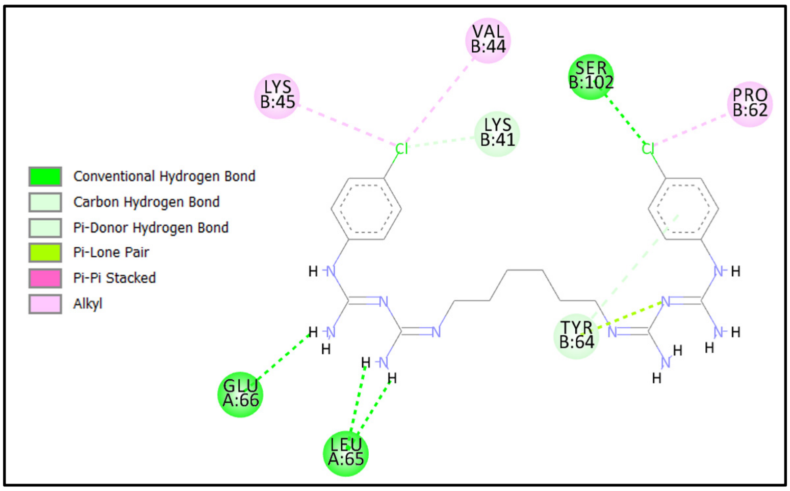

Figure S9. Interaction network between CHD and *S. sanguinis* protein structure. For clarity, the non-polar hydrogen atoms of CHD are omitted.

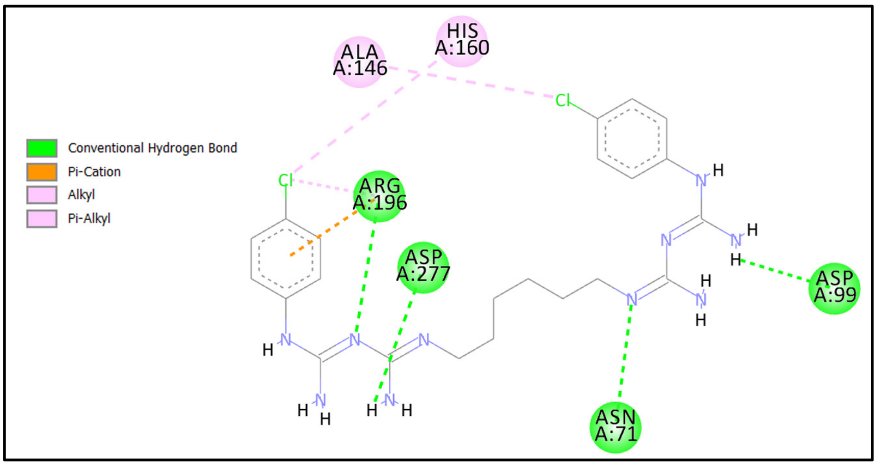

Figure S10. Interaction network between CHD and *L. paracasei* protein structure. For clarity, the non-polar hydrogen atoms of CHD are omitted.

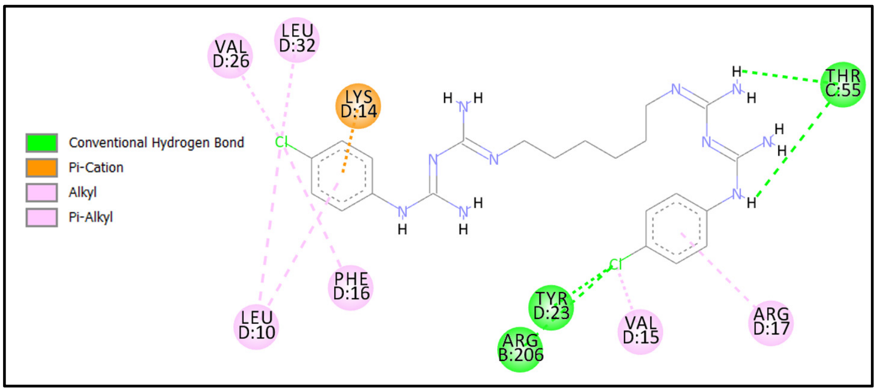

Figure S11. Interaction network between CHD and *E. faecalis* protein structure. For clarity, the non-polar hydrogen atoms of CHD are omitted.

Table S1. NMR Spectroscopic data for complexes **1** and **2** in DMSO-d_6[a].

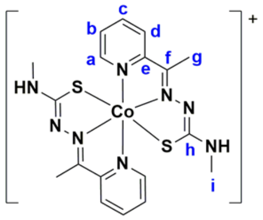

1

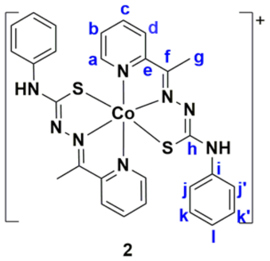

2

δ / ppm	Compounds 1	Compounds 2
a	148.7	149.2
b	126.6	127.4
c	139.9	139.9
d	124.6	125.5
e	155.4	156.7
f	157.2	158.7
g	15.0	15.8
h	178.2	175.5
i	32.1	140.1
j	-	120.4
k	-	128.7
l	-	123.3

[a] Recorded at 100 MHz for ^{13}C NMR.

Table S2. Experimental parameters used in Docking Molecular for $[Co(L^{Ph})_2]^+$ and CHD.

**Bacterial strains**	grid map	Center grid map (xyz-coordinates)
S. mutans	44 x 44 x 54	70.42, 58.59, 39.788
S. mitis	30 x 34 x 40	-2.03, -10.35, -14.478
S. sanguinis	54 x 64 x 56	-21.4, 24.385, 21.481
L. paracasei	52 x 42 x 46	6.144, 26.197, 17.86
E. faecalis	62 x 52 x 54	1.065, 2.539, 19.877

Table S3. Docking energy results and estimated inhibition constant (Ki) within the $[Co(L^{Ph})_2]^+$ and CHD with *S. mutans*, *S. mitis*, *S. sanguinis*, *L. paracasei* and *E. faecalis* bacteria.

**Bacterial strains**	ΔG $[Co(L^{Ph})_2]^+$ (kcal/mol)	Ki (HL^{Ph})	ΔG CHD[a] (kcal/mol)	Ki (CHD)
S. mutans	-7.59	2.73 uM	-8.46	1.58 uM
S. mitis	-3.45	2.98 mM	-3.03	5.97 mM
S. sanguinis	-8.66	449.53 nM	-9.49	111.36 nM
L. paracasei	-9.55	99.23 nM	-7.92	5.75 nM
E. faecalis	-8.54	555.46 nM	-11.24	634.24 nM

[a]Positive controls docked for comparison.

Table S4. Calculated H bond, pi-pi and pi-S distances of intermolecular interactions of the cobalt complex with amino acid residues of *S. mutans*, *S. mitis*, *S. sanguinis*, *L. paracasei* and *E. faecalis* bacteria.

**Complex-target (Bacterial strains)**	H bond location (length)	π- π location (length)	π -S location (length)
**$[Co(L^{Ph})_2]^+$ – *S. mutans***	N-H with O of Thr 23 (2.13 Å)	–	Ph group with Tyr 44 (4.48 Å)
**$[Co(L^{Ph})_2]^+$ – *S. mitis***	N-H with O of Asp 114 (3.19 Å)	–	Ph group with His 85 (4.44 Å)
**$[Co(L^{Ph})_2]^+$ – *S. sanguinis***	N-H with O/S of Thr 63 (1.81 Å and 3.76 Å)<br>N with OH/NH and N of Thr 15 (2.76 Å and 3.19 Å)<br>N-H with O of Met 132 (2.35 Å)	Ph group with Phe 130 (4.56 Å)	–
**$[Co(L^{Ph})_2]^+$ – *L. paracasei***	N-H with S of Arg 196 (3.18 Å)	Ph group with Phe 74 (4.74 Å and 4.78 Å)	–
**$[Co(L^{Ph})_2]^+$ – *E. faecalis***	N-H with O of Gln 241 (3.19 Å)	Ph group with Tyr 23 (4.60 Å and 4.99 Å)	Ph group with His 245 (5.11 Å)
